# Arbutin encapsulated micelles improved transdermal delivery and suppression of cellular melanin production

**DOI:** 10.1186/s13104-016-2047-x

**Published:** 2016-04-30

**Authors:** Ke Liang, Keming Xu, Dmitri Bessarab, Jonathan Obaje, Chenjie Xu

**Affiliations:** School of Chemical & Biomedical Engineering, Nanyang Technological University, 70 Nanyang Drive, Singapore, 637457 Singapore; Urah® Transdermal Pte Ltd, 51 Ubi Avenue 1, #05-13, Singapore, 408933 Singapore; NTU-Northwestern Institute for Nanomedicine, Nanyang Technological University, 50 Nanyang Avenue, Singapore, 639798 Singapore

**Keywords:** Micellar transdermal drug delivery, Micelles, Hyperpigmentation, Melanin, Arbutin

## Abstract

**Background:**

Hyperpigmentation is a skin disorder characterized by elevated production of melanin. Current treatment approaches mainly rely on the application of skin lightening chemicals, most of which have safety issues. Efficacy of delivery of the active ingredients to the target organ has also been a challenge. Transdermal based drug delivery platform has been shown to improve drug bioavailability, avoiding the hepatic first pass metabolism, decrease gastrointestinal side effects, and eventually enhance patient compliance.

**Results:**

This article explores the utilization of micellar transdermal delivery technology to improve skin penetration and efficacy of arbutin, a hyperpigmentation agent. The suppression efficacy of cellular melanin production versus cell viability of four active ingredients commonly used in skin lightening products, namely allantoin, arbutin, glycolic acid, and hyaluronic acid were first compared. Arbutin was selected for the micellar delivery studies base on its comparatively low cytotoxicity and better performance in reducing melanin production. Micellar Arbutin cream was formulated using Urah® proprietary micellar technology and was assessed for its cellular melanin suppression efficacy and skin penetration capacity.

**Conclusion:**

The results show that micellar arbutin cream improved both the delivery and cellular melanin suppression, suggesting that micellar transdermal delivery may have potential application in addressing hyperpigmentation skin disorders.

**Graphical abstract:**
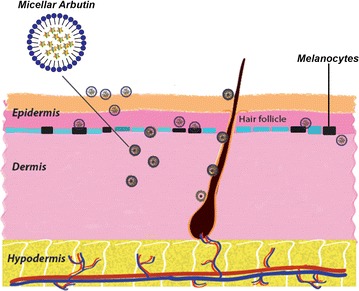
Transdermal delivery of arbutin with micelles for melanin production suppression.

**Electronic supplementary material:**

The online version of this article (doi:10.1186/s13104-016-2047-x) contains supplementary material, which is available to authorized users.

## Background

Melanin is an irregular light absorbing polymer produced by the melanocytes which are located in the basal layer of epidermis [1]. Melanin protects human skin and underlying tissues against ultraviolet (UV) rays and absorption of toxic chemicals [2]. However, excessive formation or accumulation of melanin in the skin causes hyperpigmentation skin disorders [3]. Melanin formation in skin cells can be reduced or blocked by some chemicals which are the common active ingredients in skin lightening products [4, 5]. Most of the chemicals decrease the production of melanin by suppressing tyrosinase, an enzyme governing melanin production. So that as existing skin cells are naturally exfoliated keratinocytes with less melanin come to the surface and provide a lighter skin [6, 7].

To reach the skin cells, however, the chemicals have to pass through the stratum corneum, which acts as highly resistant lipid barrier to penetration of foreign molecules into the skin [8]. Therefore in both the pharmaceutical and cosmetic fields, significant efforts have been put forth in attempts to overcome the barrier of the stratum corneum in order to deliver topically functional agents into the skin. For example, Kim disrupted the stratum corneum by using dissolving microneedle patch to deliver 4-n-butylresorcinol, a skin depigmentation agent [9]. Cell penetrating peptide conjugated liposomes were also used to enhance transdermal delivery of *Polygonum aviculare* L. extract [10]. Recently, lecithin organogel was described as a unique micellar system for the topical delivery of drugs and bioactive agents including tacrolimus, vitamins A and C, kojic acid, glycolic acid and other molecules [11, 12]. To the best of our knowledge there is no record of application of micelles for the transdermal delivery of arbutin.

In this article, we compare the suppression efficiency of cellular melanin production by four active ingredients commonly used in skin lightening products, namely allantoin, arbutin, glycolic acid, and hyaluronic acid. Due to comparatively lower cytotoxicity and higher performance in reducing melanin production, arbutin was selected from the four candidates for further studies. 5 % (w/w) arbutin was encapsulated in a micellar matrix of the cream with the view to ascertain possible effect of micellar formulation. Both the micellar and the non-micellar arbutin (containing 5 % w/w arbutin) were screened for their ability to suppress melanin production in a B16-F10 mouse melanoma cell line. Furthermore, we performed ex vivo skin penetration experiments in a Franz cell using fresh porcine skin to demonstrate the transdermal permeability of the micellar arbutin.

## Methods

Glycolic acid (≥97 %) and allantoin (≥98 %) were purchased from Fluka. Melanin, arbutin (≥98 %), hyaluronic acid (≥98 %), and Penicillin–Streptomycin were purchased from Sigma-Aldrich. phosphate buffer saline (PBS) with pH 7.4 (1×), fetal bovine serum (FBS), and Dulbecco minimum essential medium (DMEM) were purchased from Gibco. Alamar blue was purchased from Invitrogen. The porcine skin was obtained from the local supermarket and doesn’t require any ethical approval according to the national regulations. The care and use of laboratory animals used in preliminary study were monitored according to the approved protocols (BRC IACUC #151001) of the Institutional Animal Care and Use Committee (IACUC) at the Biological Resource Center (BRC) in Biopolis, Singapore.

### Cell culture

B16-F10 mouse melanoma cell line (ATCC) was maintained in DMEM containing 10 % FBS and 1 % Penicillin–Streptomycin (full medium) at 37 °C under 5 % CO_2_.

### Suppression of cellular melanin production

Four different agents (arbutin, glycolic acid, hyaluronic acid, and allantoin) were screened for their ability to suppress the melanin production through cell experiment. Briefly, B16-F10 cells were seeded at a density of 8000 cells per well in a 96 well plate 24 h before experiments. The original culture medium in the wells was then replaced with medium containing various concentrations (0.1 mg/ml, 1 mg/mg, and 5 mg/ml) of the above four agents. The concentrations were selected based on the concentration ranges usually found in commercial products (allantoin (recommended dosage is 0.5–2 %) [13, 14], arbutin (concentrations reported in the literature is 3–7 %) [15], glycolic acid (concentration reported in literature is 4–70 %) [16–18], and hyaluronic acid (concentration is up to 1 %) [19]). The serum concentration (10 %) was controlled to be the same for all wells. The culture was maintained for 5 days, during which each well was imaged every day. On day five, the media from all wells was transferred to a new plate and quantified for the absorbance at 405 nm on UV–Visible spectrophotometer (Shimadzu, UV-2450). The concentration of melanin at each well was estimated by correlating the measured absorbance with the standard curve between the melanin concentration and absorbance.

### Cell viability assay

Alamar blue assay was carried to analyze the cell viability after the incubation with various concentrations of arbutin, glycolic acid, hyaluronic acid, and allantoin. Briefly, 8000 cells were seeded per well in a 96 well plate 24 h before the experiment. Then various concentrations of arbutin, glycolic acid, hyaluronic acid, and allantoin (0.1 mg/ml, 1 mg/mg, and 5 mg/ml) were added to the wells for 24 h. Later, 10 µl of Alamar blue was directly added to the wells for 2 h. The absorbance at 600 nm was measured (Spectramax M5 Microplate/Cuvette Reader System, Molecular Devices). A series of cell dilutions were prepared to derive a standard curve between absorption and cell concentration.

### Micellar arbutin

Typically, when the cream containing functional ingredients is applied on the skin, the emulsion stability may be affected by the changes in the relative concentration of the continuous phase (solvent) on the skin environment. This presents a formulation challenge for effective transdermal products. Both the emulsion stability and the efficacy of absorption of the active ingredients may be affected by the changes in the continuous phase. The Urah^®^ micellar system used here incorporates reverse micellar transformation capability to address the change in the concentration of the continuous phase on the skin, thus enabling the micelle to work both as a solubilizer and delivery vehicle on the skin [20]. The production of arbutin-encapsulated micelles or micellar arbutin cream, was done by using the proprietary formulation method from Urah^®^ Transdermal Pte Ltd (Singapore) which comprises of the encapsulation of 5 % arbutin in a stable emulsion matrix using Urah^®^ patented bio-surfactants [20]. The non-micellar arbutin cream (cream containing 5 % arbutin without encapsulated micelles) was formulated using the standard oil-in-water emulsification approach [21].

### Suppression of cellular melanin production with micellar arbutin cream

This experiment was performed using the 24-well plate for transwell migration assay (8 µm pore, Cell Biolabs Inc.). 24 h before experiment, B16-F10 cells were seeded to the bottom chamber at a density of 8 × 10^4^ cells/well in 0.5 ml full medium. Then 100 mg of micellar arbutin cream, 100 mg of non-micellar arbutin cream (see the section above), or 5 mg of free arbutin powder was added onto the upper well. The plate was incubated at 37 °C with 5 % CO_2_ for 5 days. Every day, the cells were imaged. 5 days later, the upper wells were removed and the absorbance of the media at the bottom chamber at 405 nm was measured with Spectramax M5 Microplate/Cuvette Reader System (Molecular Devices). The blank cream containing no arbutin was used as a control.

### Ex vivo skin penetration study with Franz cell

Fresh porcine skin (abdominal skin) was used in the ex vivo skin permeation studies. The hair and subcutaneous fat were carefully removed before experiment. The porcine skin was placed and clamped between the donor and receptor compartments of a Franz cell assembly with the *stratum corneum* of the skin piece facing the donor compartment. The receiving compartment was filled with PBS, avoiding the air bubbles between the underside of the skin and the PBS solution. 1 g of micellar arbutin cream, 1 g of non-micellar arbutin cream or the blank cream (no arbutin) was applied homogeneously onto the stratum corneum of the skin. A magnetic stirrer was put into the bottom of Franz cell, which stirred continuously at 400 rpm. The PBS solution was sampled (100 µl) at 1, 2, 4, 8, 12 and 24 h respectively. Same volume of PBS was refilled back to the Franz cell each time after sampling.

The transmigrated arbutin was quantified by high performance liquid chromatography (HPLC) (Agilent 1100 HPLC VWD) on a LC column (Poroshell, 120 EC-C18, 4.6 × 100 mm, 2.7 µm) and detected at 222 nm. The mobile phase was a mixture of water:methanol:0.1 M hydrochloric acid (89:10:1, v/v/v) with 0.5 ml/min flow rate. The area of the arbutin peak was measured and converted to the concentration based on the standard curve.

### Statistical analysis

All statistics were performed with the Origin Pro 8.0 (OriginLab). The data were analyzed by one-way ANOVA. *P < 0.05, **P < 0.01 were considered to indicate a statistically significant difference.

## Results and discussion

### Melanin suppression activity and cytotoxicity

Allantoin, arbutin, glycolic acid, and hyaluronic acid were firstly compared for their suppression of melanin production in B16-F10 mouse melanoma cells. The cells were incubated with these chemicals under four concentrations (0, 0.1, 1 and 5 mg/ml) for 5 days. At day 5, the cell medium was transferred to a new plate and the secreted melanin concentrations for each well were quantified by measuring the absorption at 405 nm and correlating the results to the concentration-UV absorption standard curve (Additional file 1: Figure S1). The viability of cells left in the original plate were analysed using alamar blue assay.

**Fig. 1:**
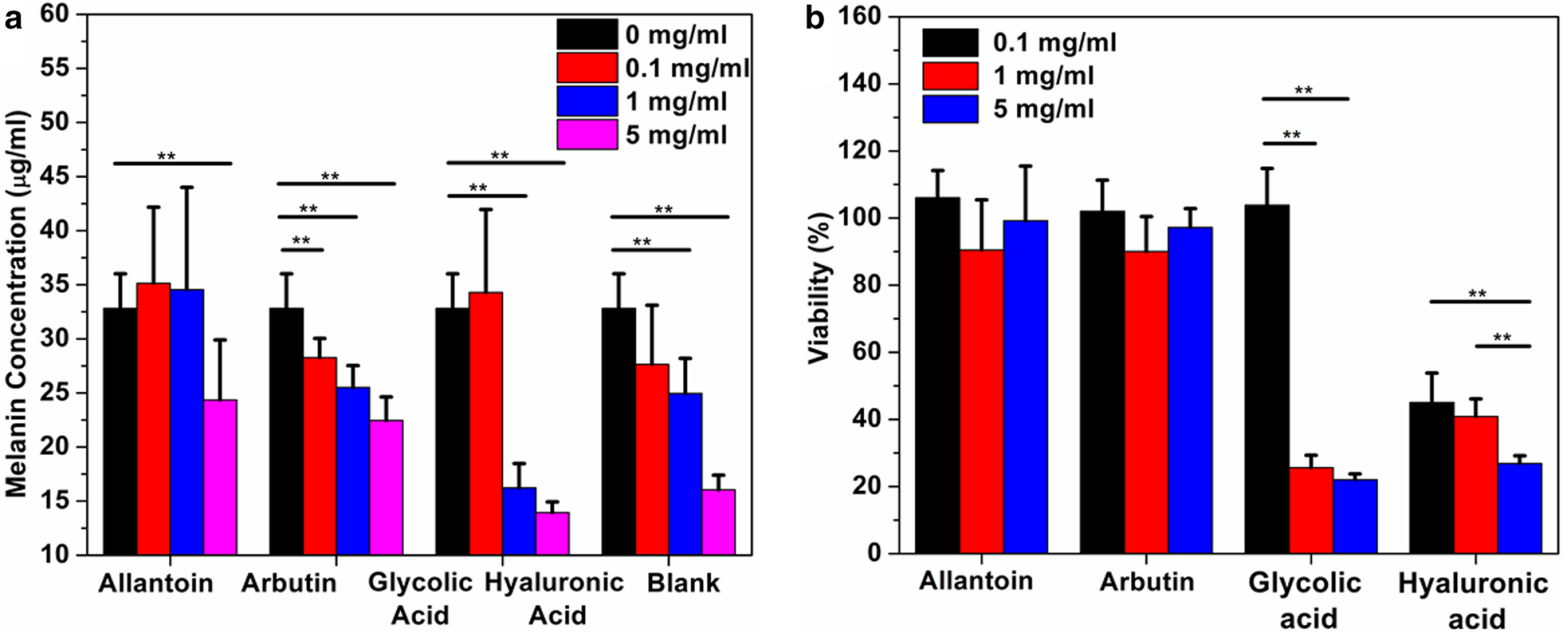
Comparison of allantoin, arbutin, glycolic acid, and hyaluronic acid: **a** suppression of melanin production at 0.1, 1, and 5 mg/ml in a 5-day culturing. **b** Cell quantification after a 5-day culturing. *P < 0.05, **P < 0.01, as determined by one-way ANOVA

Based on the quantification of secreted melanin (Fig. 1a), glycolic acid had the strongest effect in inhibiting the melanin production at the 1 and 5 mg/ml concentrations whereas allantoin was the least effective at both 1 and 5 mg/ml. However, the cell quantification (Fig. 1b) revealed that both glycolic acid and hyaluronic acid were cytotoxic. The remaining numbers of cells in the wells after treatment with 1 and 5 mg/ml glycolic acid represented only 20–30 % of numbers of cells detected in the control wells. The daily imaging suggested that the lower melanin amounts observed for glycolic and hyaluronic acids were due to the decrease of cell viability (Additional file 1: Figure S2). In comparison, both allantoin and arbutin did not show any influence over the cell viability and proliferation at the three concentrations used.

Given the higher cell viability and relatively effective inhibition of cellular melanin production, arbutin was chosen for further micellar formulation studies.

### Micellar arbutin is a formulation with active arbutin

The cream with micelle encapsulated arbutin (or micellar arbutin cream) with 5 % arbutin (w/w) was prepared as described in the experimental section. This was compared with the non-micellar arbutin cream. Through dynamic light scattering study (Additional file 1: Figure S3), the micellar arbutin was around 90 nm, which was close to the size of blank micelle (70 nm). If the arbutin was not encapsulated within the micelles, the particle size was around 250 nm.

**Fig. 2:**
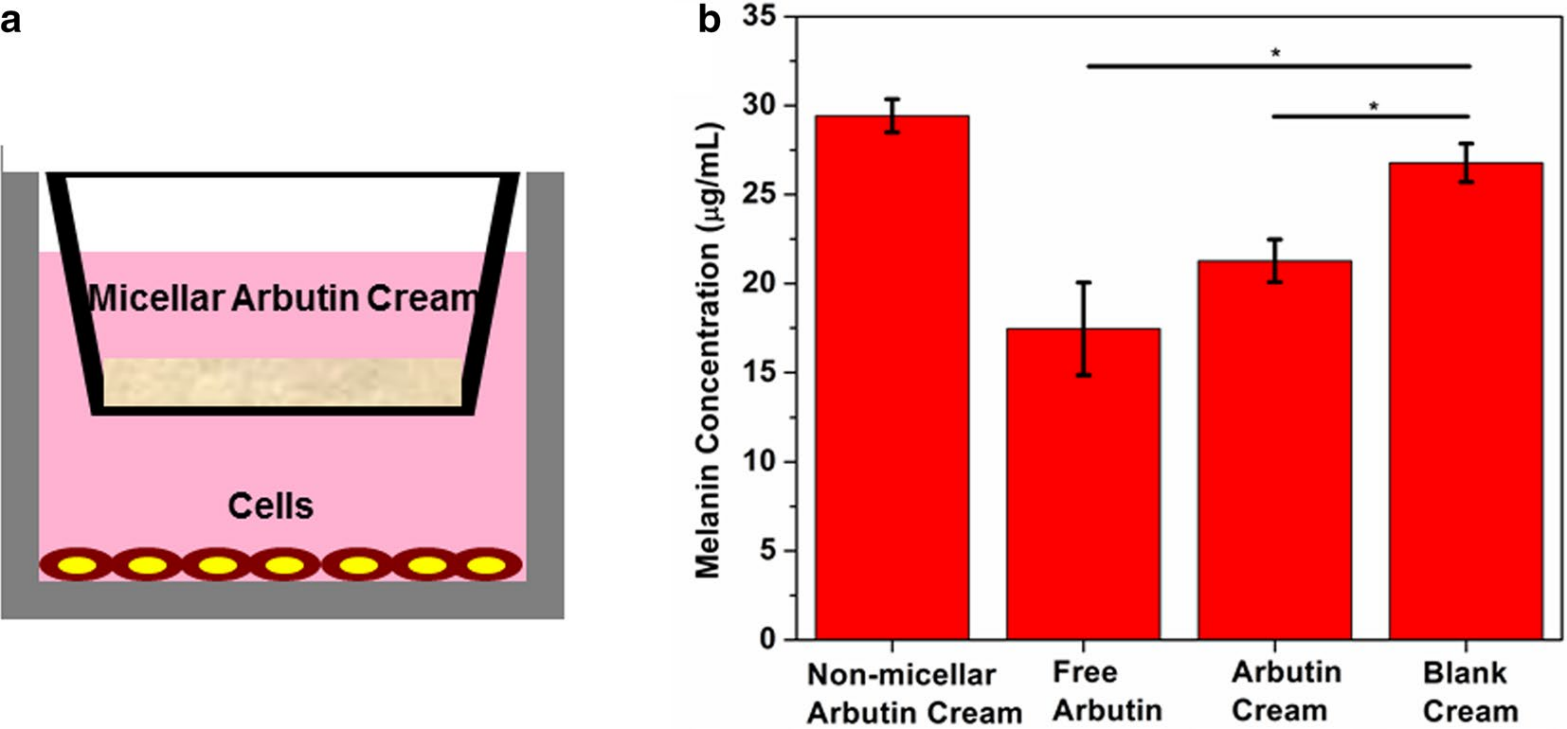
Micellar arbutin cream for the suppression of cellular melanin production: **a** illustration of the transwell experiment. **b** Melanin quantification after a 5-day culturing. Results represent the mean ± standard deviation of three independent cultures and determinations. *P < 0.05 compared with the sample treated with blank cream, as determined by one-way ANOVA

To test whether the micellar arbutin retains its original melanin-suppression ability, we co-cultured the B16-F10 cells with non-micellar arbutin cream, free arbutin, micellar arbutin cream, and blank cream in a transwell (Fig. 2a). Transwell was used to prevent the physical contact between cream and cells. We expected that the melanin suppression efficiency of micellar arbutin cream would be somewhat lower in comparison to free arbutin, since the micelles with arbutin had to leave the cream phase before they got into the medium with cells. Based on the comparative melanin concentrations, addition of free arbutin reduced melanin concentration by 40 % (relatively to the blank cream), micellar arbutin reduced melanin concentration by about 25 %, whereas non-micellar arbutin by 10 % (Fig. 2b). In addition, we did not see any significant cytotoxicity of micellar arbutin in the 5-days long experiment. Cells in the wells with free arbutin, micellar arbutin, non-micellar arbutin, and blank cream grew and proliferated at similar rates (Additional file 1: Figure S3). Thus, the micellar arbutin cream has strong advantage in the suppression of melanin production while retaining its safety credentials.

### Micellar arbutin cream shows enhanced transdermal activity

**Fig. 3:**
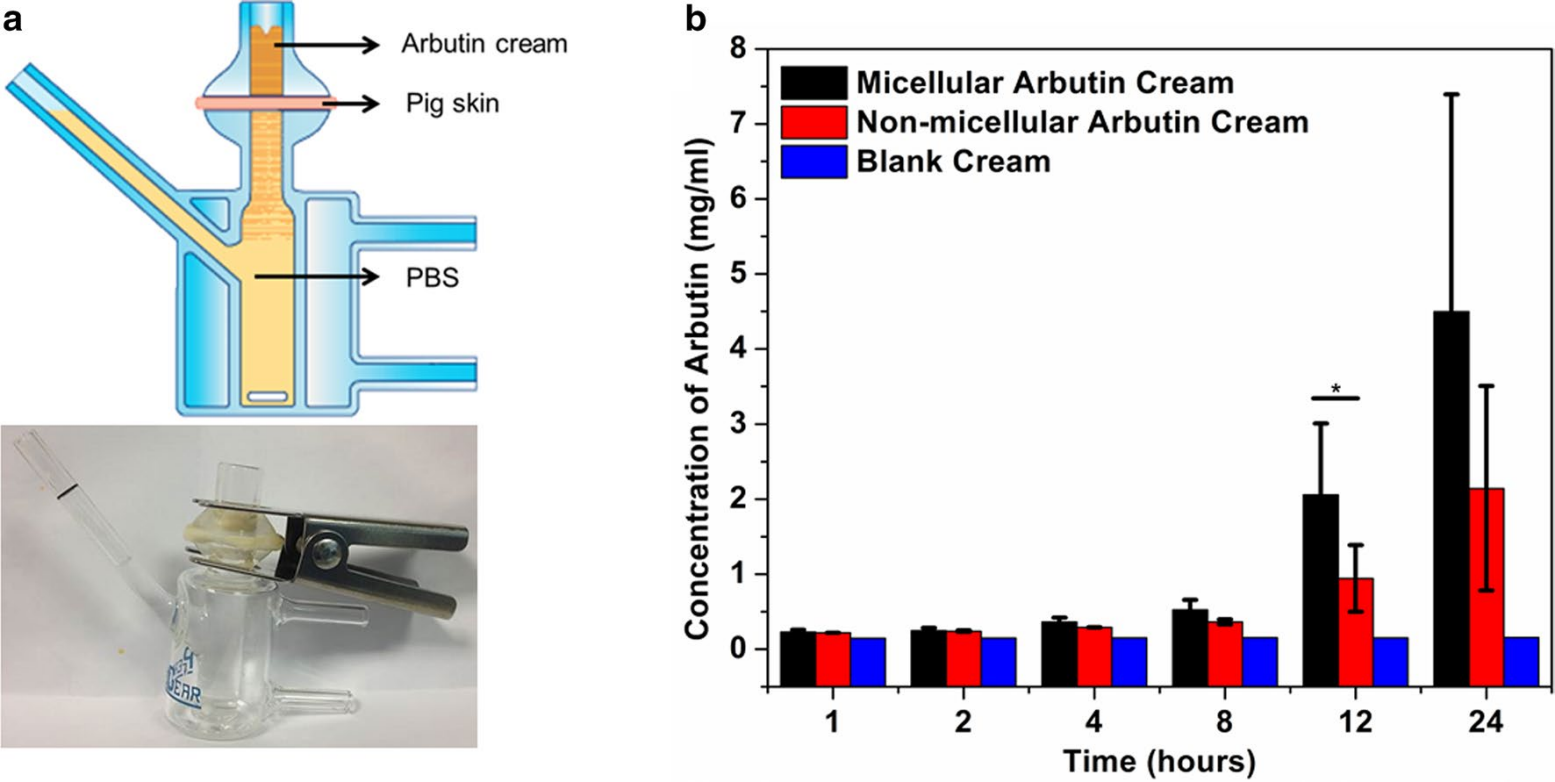
Ex vivo skin penetration experiment: **a** illustration and a photo of Franz cell experiment. **b** Quantification of arbutin in the receiving well during an 24-h incubation. *P < 0.05, **P < 0.01, as determined by one-way ANOVA

We examined the skin penetration capacity of micellar arbutin in a static Franz diffusion cell (Fig. 3a). Fresh pig skin after the removal of fat and hair was used due to its similarity to human skin [22]. The transmigrated arbutin in the receiving well was quantified by HPLC (Additional file 1: Figure S4). The area of arbutin peak, identified with standard samples was quantified and converted to the arbutin concentration by correlating with the standard curve (Additional file 1: Figure S5). As shown in Fig. 3b, there was a slight increase in the concentration of arbutin in the receiving well after 1 h for both micellar arbutin cream and non-micellar arbutin cream, in comparison to the blank cream. This suggests that same level of passive diffusion was taking place in both non-micellar arbutin at the initial stage. Four hours upon application, the micellar arbutin cream showed gradual increase in the concentration of arbutin delivered through the skin in comparison with non-micellar arbutin cream. The difference nearly doubled after 12 h. These data suggest that micellar arbutin cream can improve the steady delivery of arbutin through the skin over a long period of time.

## Conclusion

Hyperpigmentation is the result of excessive melanin production by the pigment cells—melanocytes located in the basal layer of the epidermis. Existing treatment approaches use topically applied chemicals which activity either enhances the epidermal cell turn-over due to the peeling or exerts inhibitory action on the melanin synthesis machinery. The latter requires the penetration of skin barrier presented by *stratum corneum* and further diffusion of the active substance through other epidermal layers to target melanocytes in the basal layer. An efficient transdermal delivery system would increase the treatment efficacy and reduce the potential cytotoxicity of the active ingredient [5].

The aim of the present study is to evaluate the relative safety and inhibitory effect of four chemical compounds used in skin care and hyperpigmentation treatment products, and to further evaluate possible effects of micellar encapsulation on the most effective and least toxic candidate.

We compared the in vitro capacity of arbutin, glycolic acid, hyaluronic acid and allantoin to inhibit melanin synthesis while accessing their cytotoxicity (Fig. 1). It turns out that arbutin was most effective in suppressing the cellular melanin production while having the lowest cytotoxicity.

Previously, the micellar formulation has been used for transdermal delivery of glucosamine to treat osteoarthritis, and improved Ritchie Articular Index pain score from the baseline (day 0) in all demographic group and further enhanced functional abilities were evidenced [23]. Our preliminary data indicate that the glucosamine concentration in the mouse blood 2 h after the application of micellar glucosamine cream was about 10 times higher than that observed for oral delivery (Additional file 1: Figure S7). Here, arbutin was formulated with this technology and the activity of arbutin was retained in the formulation, about 80 % as effective as free arbutin (Fig. 2).

Finally, the ex vivo skin penetration experiment demonstrated that the micellar arbutin cream possesses enhanced transdermal functionality (Fig. 3). During the first 4 h of the experiment the levels of arbutin penetrated through the skin were similar for the micellar arbutin cream and non-micellar arbutin cream. However, the level rose dramatically thereafter for micellar arbutin compared to the non-micellar arbutin cream.

Taken together our data provide evidence that micellar arbutin formulation could improve arbutin delivery and thus enhance the suppression of melanin synthesis. Micellar arbutin therefore has the potential to be developed into transdermal delivery system to address hyperpigmentation skin disorders.

## Additional file

10.1186/s13104-016-2047-x **Figure S1.** Concentration-UV absorption relationship of melanin. **Figure S2.** Daily imaging of B16-F10 mouse melanoma cells during a 5-day culturing with allantoin, arbutin, glycolic acid, or hyaluronic acid at various concentrations. **Figure S3.** Photos of diluted PBS solution of micellar arbutin cream (MA), blank cream (BM), and non-micellar arbutin cream (NMA) and their Hydrodynamic diameters. **Figure S4.** Daily imaging of B16-F10 mouse melanoma cells during a 5-day culturing with blank cream, free arbutin, micellar arbutin cream, and non-micellar arbutin cream. **Figure S5.** HPLC analysis of arbutin in the receiving well during the skin penetration experiment at different hours. **Figure S6.** Relationship between the arbubin concentrations and peak area in the HPLC measurement. **Figure S7.** Concentration of glucosamine in blood plasma upon oral administration of glucosamine solution and topical application of URAH micellar glucosamine cream.

## Abbreviations

UV: ultraviolet
PBS: phosphate buffer saline
FBS: fetal bovine serum
DMEM: Dulbecco minimum essential medium
HPLC: high performance liquid chromatography
