# Supplementary material for: Arbutin encapsulated micelles improved transdermal delivery and suppression of cellular melanin production
Source: BMC Res Notes. 2016 Apr 30;9:254. doi: 10.1186/s13104-016-2047-x (PMC4851826; doi:10.1186/s13104-016-2047-x)
Supplement: Supplementary file 1 — 10.1186/s13104-016-2047-x Figure S1. Concentration-UV absorption relationship of melanin. Figure S2. Daily imaging of B16-F10 mouse melanoma cells during a 5-day culturing with allantoin, arbutin, glycolic acid, or hyaluronic acid at various concentrations. Figure S3. Photos of diluted PBS solution of micellar arbutin cream (MA), blank cream (BM), and non-micellar arbutin cream (NMA) and their Hydrodynamic diameters. Figure S4. Daily imaging of B16-F10 mouse melanoma cells during a 5-day culturing with blank cream, free arbutin, micellar arbutin cream, and non-micellar arbutin cream. Figure S5. HPLC analysis of arbutin in the receiving well during the skin penetration experiment at different hours. Figure S6. Relationship between the arbubin concentrations and peak area in the HPLC measurement. Figure S7. Concentration of glucosamine in blood plasma upon oral administration of glucosamine solution and topical application of URAH micellar glucosamine cream. [file 13104_2016_2047_MOESM1_ESM.docx]

Supporting Information for

Arbutin Encapsulated Micelles Improved Transdermal Delivery and Suppression of Cellular Melanin Production

Ke Liang^1^, Keming Xu^1^, Dmitri Bessarab^2^, Jonathan Obaje^2^, Chenjie Xu^1,3*^

^1^ School of Chemical & Biomedical Engineering, Nanyang Technological University, Singapore 637457

^2^ Urah® Transdermal Pte Ltd, 18 Nanyang Drive, Singapore 637723

^3^ NTU-Northwestern Institute of Nanomedicine, Nanyang Technological University, 50 Nanyang Avenue, Singapore 639798

* Correspondence: [cjxu@ntu.edu.sg](mailto:cjxu@ntu.edu.sg)


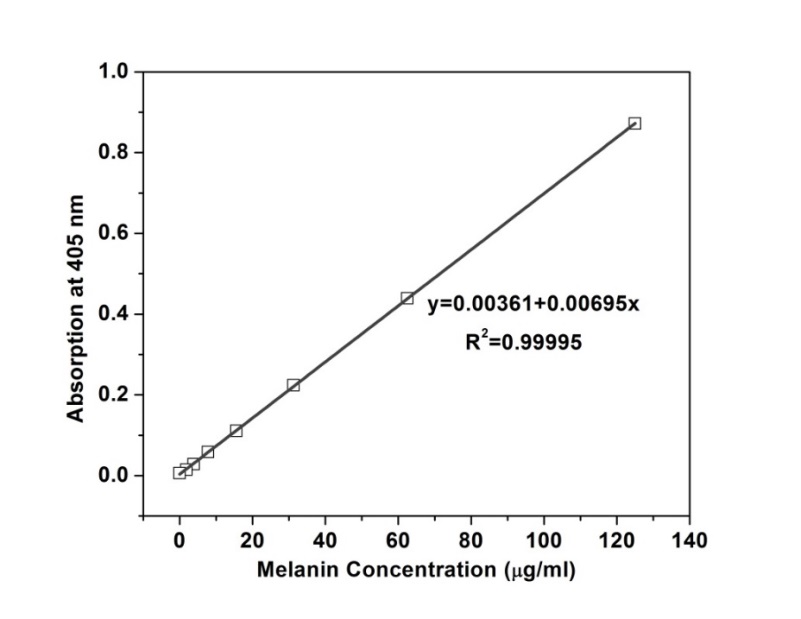


**Figure S1**. Concentration-UV absorption relationship of melanin.


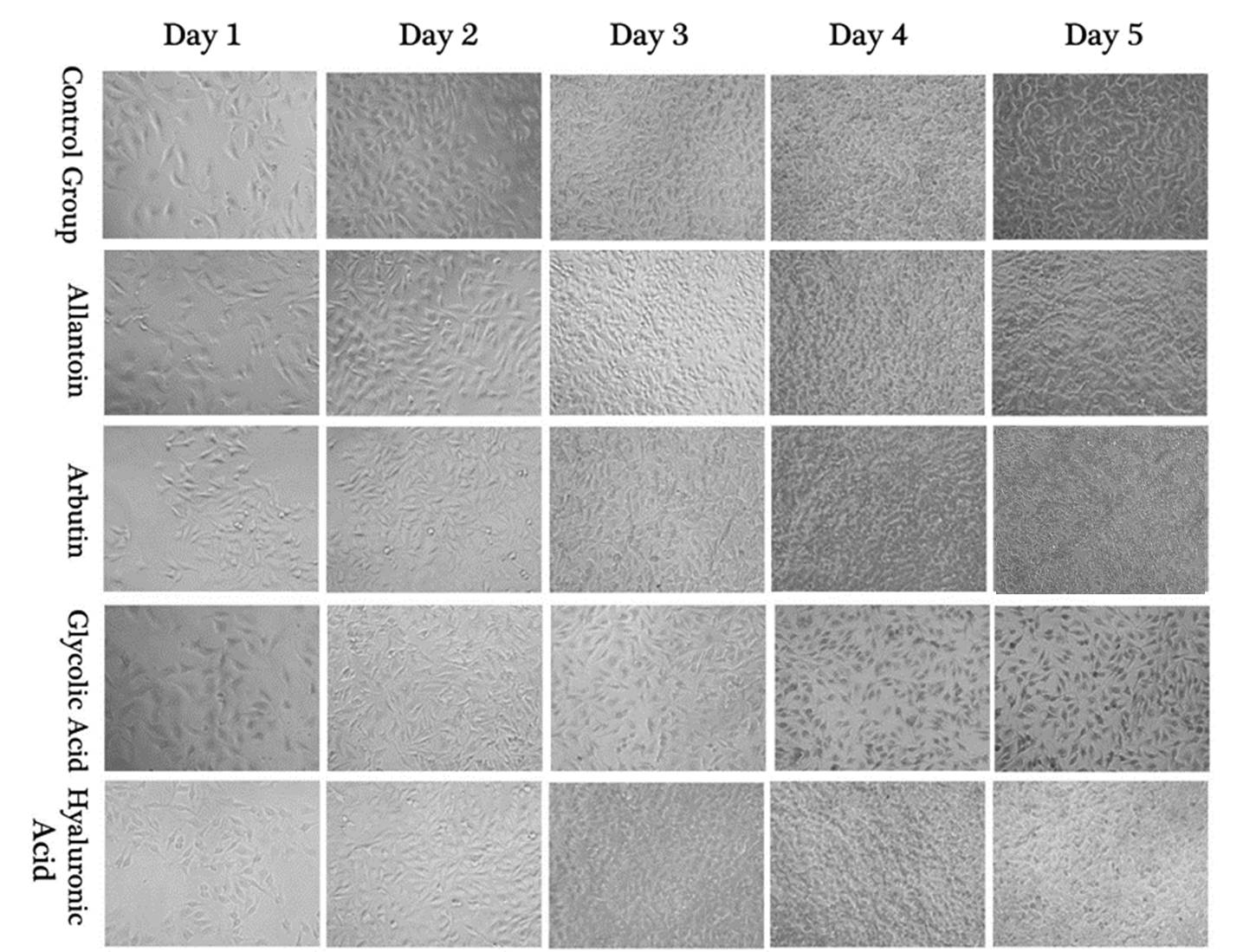


**Figure S2**. Daily imaging of B16-F10 mouse melanoma cells during a 5-day culturing with allantoin, arbutin, glycolic acid, or hyaluronic acid at various concentrations.


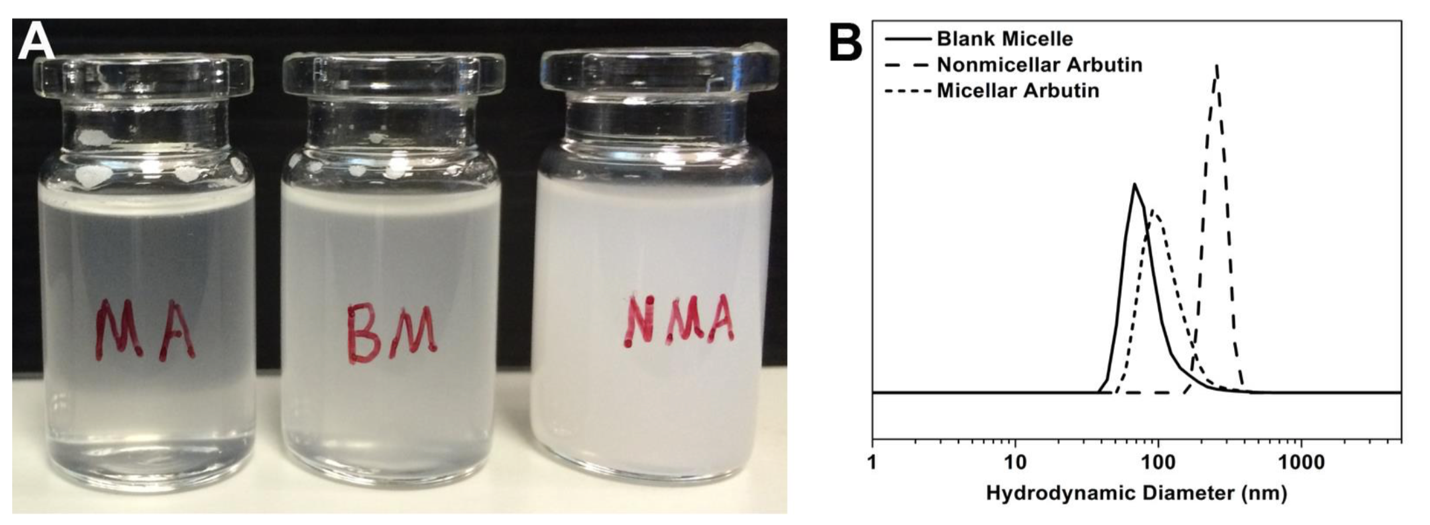


**Figure S3**. (A) Photos of diluted PBS solution of micellar arbutin cream (MA), blank cream (BM), and non-micellar arbutin cream (NMA); (B) Hydrodynamic diameter of micellar arbutin cream, blank cream, and non-micellar arbutin cream measured through dynamic light scattering.

**Day1 Day2 Day 3 Day 4 Day 5**

**Blank cream**

**Free arbutin**

**Micellar**

**arbutin**

**Non-micellar**

**arbutin**


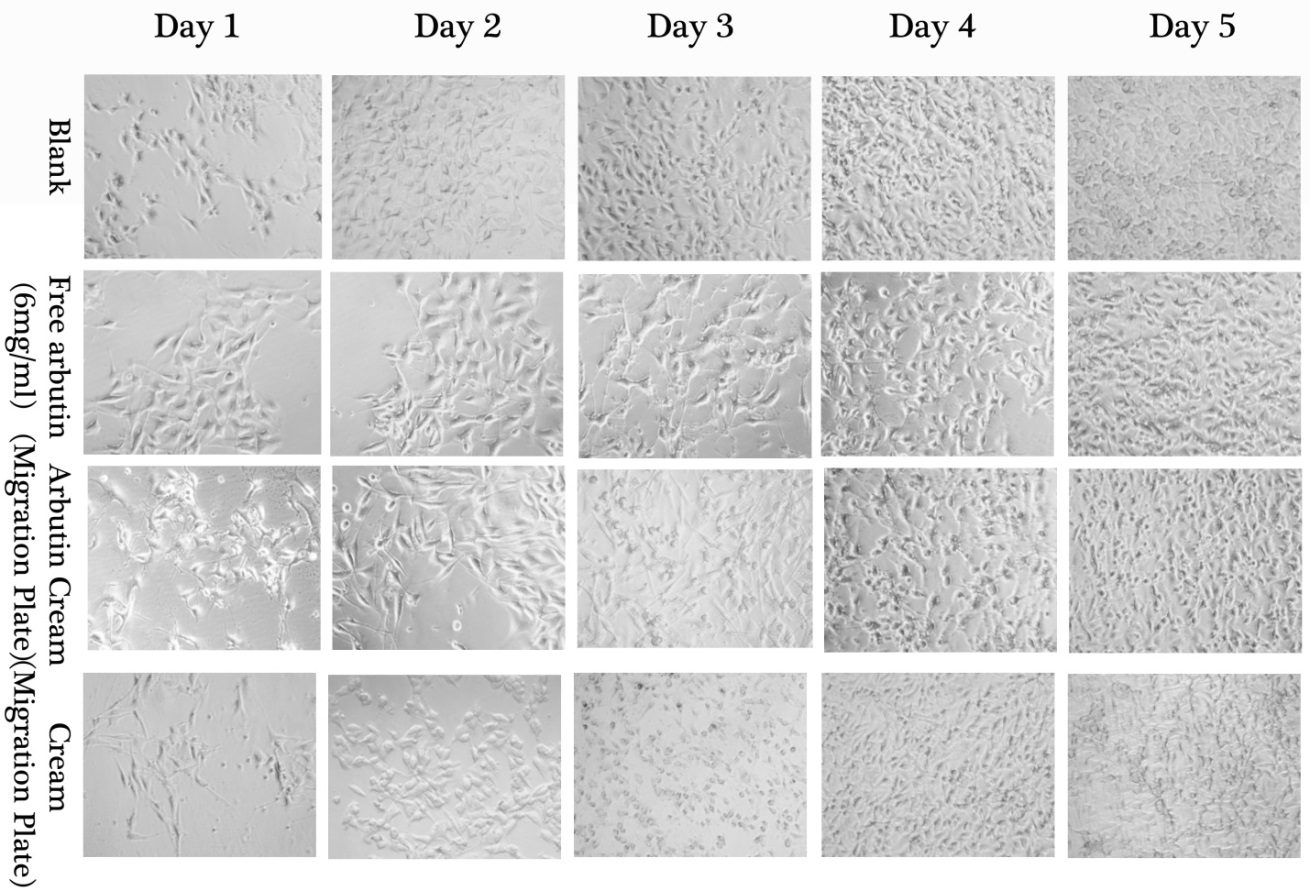


**Figure S4**. Daily imaging of B16-F10 mouse melanoma cells during a 5-day culturing with blank cream, free arbutin, micellar arbutin cream, and non-micellar arbutin cream


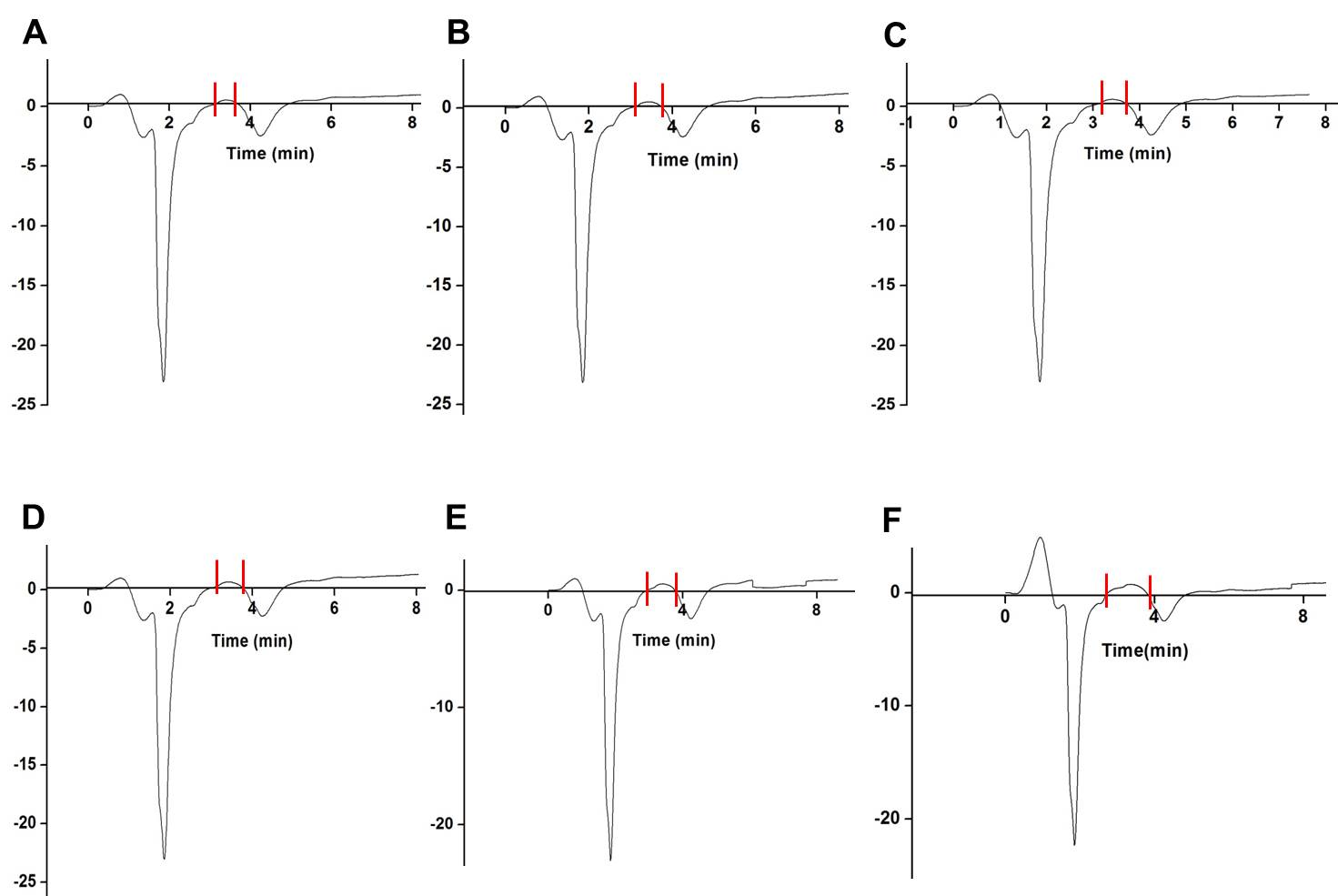


**Figure S5**. HPLC analysis of arbutin in the receiving well during the skin penetration experiment at (**A**) 0.5 hour, (**B**) 1 hour, (**C**) 2 hours, (**D**) 6 hours, (**E**) 12 hours, and (**F**) 24 hours. The peak area between two red lines represents arbutin.


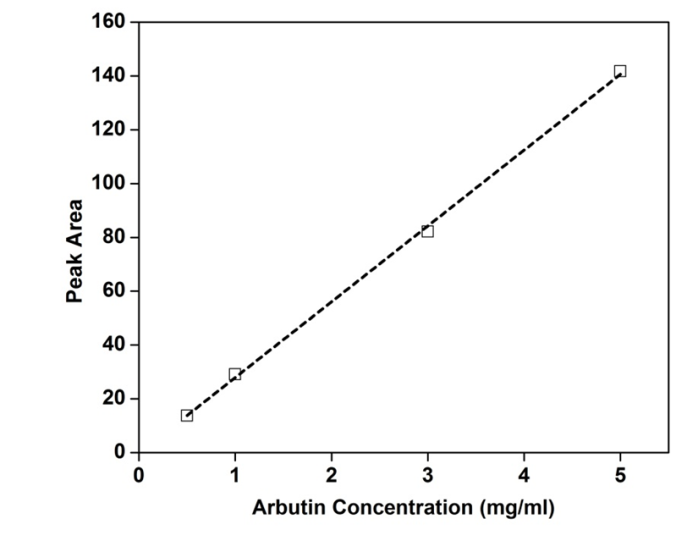


**Figure S6.** Relationship between the arbubin concentrations and peak area in the HPLC measurement

**Preliminary data**

***Determination of plasma concentration of glucosamine in mice after the topical application of micellar cream and oral administration of water solution***

The animal study was conducted at Biological Resource Centre (BRC) A*STAR Singapore. The experiments were carried out in accordance with the Institutional Animal Care and Use Committee (IACUC) protocol number BRC IACUC #151001, which was approved by IACUC of BRC A*STAR.

In transdermal delivery, mice were shaved at the dorsal region 24 hours before the experiment. The micellar cream containing 8% glucosamine was applied once (glucosamine sulphate dosage of 0.4 g per kg of body weight) by gentle massage for 30 seconds into the skin on the shaved area of about 9 cm^2^ located on the animal back. The site of cream application was left uncovered.

For oral administration, glucosamine sulphate was used as a 4% solution in distilled water (40 mg of dry powder of glucosamine sulphate/ml) and gavaged at the dosage of 0.4g/kg of body weight.

The blood sample were collected from the tail vein into EDTA containing tubes at time intervals. Blood samples collected prior to glucosamine treatment (0 hrs) served as controls. The plasma concentrations of glucosamine following oral and topical cream applications were determined using HPLC technique.

**Figure S7.** Concentration of glucosamine in blood plasma upon oral administration of glucosamine solution and topical application of URAH micellar glucosamine cream.
